# A Role for Non-Covalent SUMO Interaction Motifs in Pc2/CBX4 E3 Activity

**DOI:** 10.1371/journal.pone.0008794

**Published:** 2010-01-20

**Authors:** Jacqueline C. Merrill, Tiffany A. Melhuish, Michael H. Kagey, Shen-Hsi Yang, Andrew D. Sharrocks, David Wotton

**Affiliations:** 1 Department of Biochemistry and Molecular Genetics and Center for Cell Signaling, University of Virginia, Charlottesville, Virginia, United States of America; 2 Faculty of Life Sciences, University of Manchester, Manchester, United Kingdom; Ohio State University, United States of America

## Abstract

**Background:**

Modification of proteins by the small ubiquitin like modifier (SUMO) is an essential process in mammalian cells. SUMO is covalently attached to lysines in target proteins via an enzymatic cascade which consists of E1 and E2, SUMO activating and conjugating enzymes. There is also a variable requirement for non-enzymatic E3 adapter like proteins, which can increase the efficiency and specificity of the sumoylation process. In addition to covalent attachment of SUMO to target proteins, specific non-covalent SUMO interaction motifs (SIMs) that are generally short hydrophobic peptide motifs have been identified.

**Methodology/Principal Findings:**

Intriguingly, consensus SIMs are present in most SUMO E3s, including the polycomb protein, Pc2/Cbx4. However, a role for SIMs in SUMO E3 activity remains to be shown. We show that Pc2 contains two functional SIMs, both of which contribute to full E3 activity in mammalian cells, and are also required for sumoylation of Pc2 itself. Pc2 forms distinct sub-nuclear foci, termed polycomb bodies, and can recruit partner proteins, such as the corepressor CtBP. We demonstrate that mutation of the SIMs in Pc2 prevents Pc2-dependent CtBP sumoylation, and decreases enrichment of SUMO1 and SUMO2 at polycomb foci. Furthermore, mutational analysis of both SUMO1 and SUMO2 reveals that the SIM-interacting residues of both SUMO isoforms are required for Pc2-mediated sumoylation and localization to polycomb foci.

**Conclusions/Significance:**

This work provides the first clear evidence for a role for SIMs in SUMO E3 activity.

## Introduction

Covalent modification of proteins by SUMO is an essential process in mammals, as evidenced by the embryonic lethality of mouse mutants lacking the SUMO conjugating enzyme, Ubc9 [1]. SUMO modification regulates numerous nuclear processes, including transcription, DNA replication and repair, and chromosome segregation; and also plays a role in the transport of proteins through the nuclear pore [2], [3], [4]. SUMO is a member of the larger family of ubiquitin like proteins, which shares about 18% sequence identity to ubiquitin, and is structurally quite similar [4], [5], [6], [7]. Like ubiquitin, SUMO is covalently attached to lysine residues within the target protein, although in the majority of cases SUMO is attached to a lysine within the ψKxE consensus site (where ψ is hydrophobic and x is any residue) [8], [9]. In mammals there are four SUMO isoforms, including SUMO1, which most closely resembles the single yeast Smt3. SUMO2 and -3, which are very similar to each other, contain an internal sumoylation consensus site and more readily form poly-SUMO chains [10], [11]. SUMO4, variants of which have been linked to diabetes, is more similar to SUMO2 and 3 than SUMO1 [12]. Attachment of SUMO to lysine residues within a target protein is mediated by a conserved enzymatic pathway [4], [5], [13]. SUMO is first cleaved at a di-glycine motif close to the carboxyl-terminus, to generate the mature form of the protein. SUMO processing is carried out by members of a family of SUMO proteases, termed SENPs (for SUMO/sentrin specific peptidase), that can also catalyze the removal of SUMO from lysines within target proteins [14], [15]. The processed SUMO is transferred from the heterodimeric E1 enzyme to Ubc9, which is the sole SUMO E2 conjugating enzyme. The loading of Ubc9 with SUMO is the only energy requiring step in the modification pathway, and results in the attachment of SUMO to the catalytic cysteine of Ubc9 via a thioester linkage. SUMO-loaded Ubc9 can modify substrate proteins directly, resulting in the covalent attachment of SUMO to the acceptor lysine. Although there is no absolute requirement for an E3, a number of SUMO E3s have been identified, including members of the PIAS (protein inhibitor of activated STAT) family, the polycomb protein, Pc2/Cbx4, and RanBP2/Nup358 [16], [17], [18], [19]. SUMO E3s interact with both E2 and substrate and promote the transfer of SUMO from Ubc9 to the target lysine in the substrate.

In addition to the covalent attachment of SUMO to lysine residues in target proteins, recent work has identified specific motifs that mediate non-covalent interactions with SUMO [20], [21], [22], [23]. In general these motifs consist of a hydrophobic core, which is often flanked by acidic residues. The best characterized of the SUMO interaction motifs (SIMs) have the consensus sequence, V/I-x-V/I-V/I or V/I-V/I-x-V/I/L, where position two or three can be any amino acid [22], [23]. Structural studies have demonstrated that these hydrophobic SIMs bind to the second β-strand and the first α helix of SUMO [22], [23], [24]. SIMs can bind to SUMO in two opposite orientations, and the presence of acidic residues either upstream or downstream of the hydrophobic core has been suggested to contribute to the orientation of binding by forming interactions with basic residues in SUMO [23]. With increased attention focused on the non-covalent interaction of SUMO with SIM containing proteins, it has become clear that there is also some flexibility in the precise sequence of the hydrophobic core of the SIM. For example, the corepressor CoREST1 binds to SUMO2, but not SUMO1, via a SIM, with a five amino acid core in which positions 1, 3 and 5 are hydrophobic residues [25]. Additionally, it has been shown that CK2-mediated phosphorylation of serine residues surrounding the hydrophobic core of the PIAS1 SIM can increase SUMO binding [26]. Clearly, the somewhat variable nature of the consensus SIM, combined with the fact that such a short amino acid sequence will be found by chance quite frequently, emphasizes the importance of testing the functionality of potential SIMs.

Interestingly, consensus SIMs are found in several components of the sumoylation machinery, including a subunit of the E1 heterodimer, members of the PIAS E3 family, RanBP2/Nup358, and Pc2. Additionally, SIMs are found in some SUMO substrates. This clearly raises the possibility that components of the modification pathway interact non-covalently with SUMO to facilitate its transfer to substrates. In support of this, the SIM in RanBP2/Nup358 is directly adjacent to the minimal IR1-IR2 domain that has E3 activity. However, although this SIM has been shown to bind to SUMO [22], it appears not to be essential for E3 activity, at least *in vitro*. Interestingly, analysis of the SIM in PIAS1 demonstrated that it can indeed bind to both SUMO1 and SUMO2, and that the SIM is required for the transcriptional regulatory activity of PIAS1 [26]. However, the PIAS1 SIM has not been shown to play any role in the ability of PIAS1 to act as a SUMO E3. For some substrates, including BLM, USP25 and Daxx, it appears that the presence of a SIM within the substrate itself can increase sumoylation by facilitating the recruitment of SUMO-loaded Ubc9, via non-covalent interaction of the Ubc9-bound SUMO with the SIM [27], [28], [29]. This promotes binding of Ubc9 to the target modification site in the substrate, allowing for transfer of the SUMO to the substrate. In addition to playing possible roles in SUMO modification, SIMs may also act as protein interaction modules which facilitate the formation of multi-protein complexes. The importance of sumoylation for the maintenance of PML bodies has been known for some years [30], [31]. However, it has now been shown that the presence of a SIM in the PML protein is also required for the recruitment of sumoylated proteins to PML bodies, and for PML body formation [32]. Other proteins, such as the Ring finger ubiquitin E3, RNF4, have multiple SIMs which appear to facilitate the binding of poly-sumoylated proteins. The binding of RNF4 to sumoylated proteins, including PML, allows for the RNF4 mediated ubiquitylation of the sumoylated substrate protein [33], [34], [35], [36].

The polycomb protein, Pc2/Cbx4, is a component of the PRC1 (polycomb repressive complex) complex, and was first identified based on similarity with the *Drosophila* Pc protein [37], [38]. In flies there is a single Pc, whereas mammals have five Cbx paralogs, in addition to the HP1 proteins [39]. Outside the conserved amino-terminal chromodomains there is relatively little sequence similarity between *Drosophila* Pc and mammalian Cbx proteins, except for a short hydrophobic region at the carboxyl-termini of all but the HP1 proteins. We have shown that Pc2 is a SUMO E3 for the transcriptional corepressors CtBP1 and CtBP2 [18], [40], and several other proteins have been identified as substrates for Pc2 E3 activity, including the kinase, HIPK2, the transcriptional regulator, SIP1, a DNA methyltransferase and the CTCF insulator protein [41], [42], [43], [44]. Structure function analyses have revealed at least two domains that contribute to Pc2 E3 activity, and it is intriguing to note that each of these two sub-domains contains a single consensus SIM [39], [40]. However, the functional relevance of the SIMs in Pc2 has not been tested directly.

*In vitro* Pc2 appears to have quite weak E3 activity, whereas its E3 activity in transfected cells, at least for CtBP, is quite robust [18], [40]. We, therefore, decided to analyze the *in vivo* contributions of the Pc2 SIMs to its E3 activity. Here we show that both SIMs in Pc2 contribute to non-covalent SUMO binding and are required for full E3 activity, as well as for covalent modification of Pc2 itself by SUMO. Analysis of mutant forms of both Pc2 and SUMO1 or SUMO2 demonstrates that recruitment of SUMO to Pc2 containing sub-nuclear foci requires non-covalent SUMO-SIM interactions. This work provides clear evidence that SUMO-SIM interactions are required for the E3 activity of Pc2.

## Results

### Pc2 Contains Two SIMs

**Figure 1:**
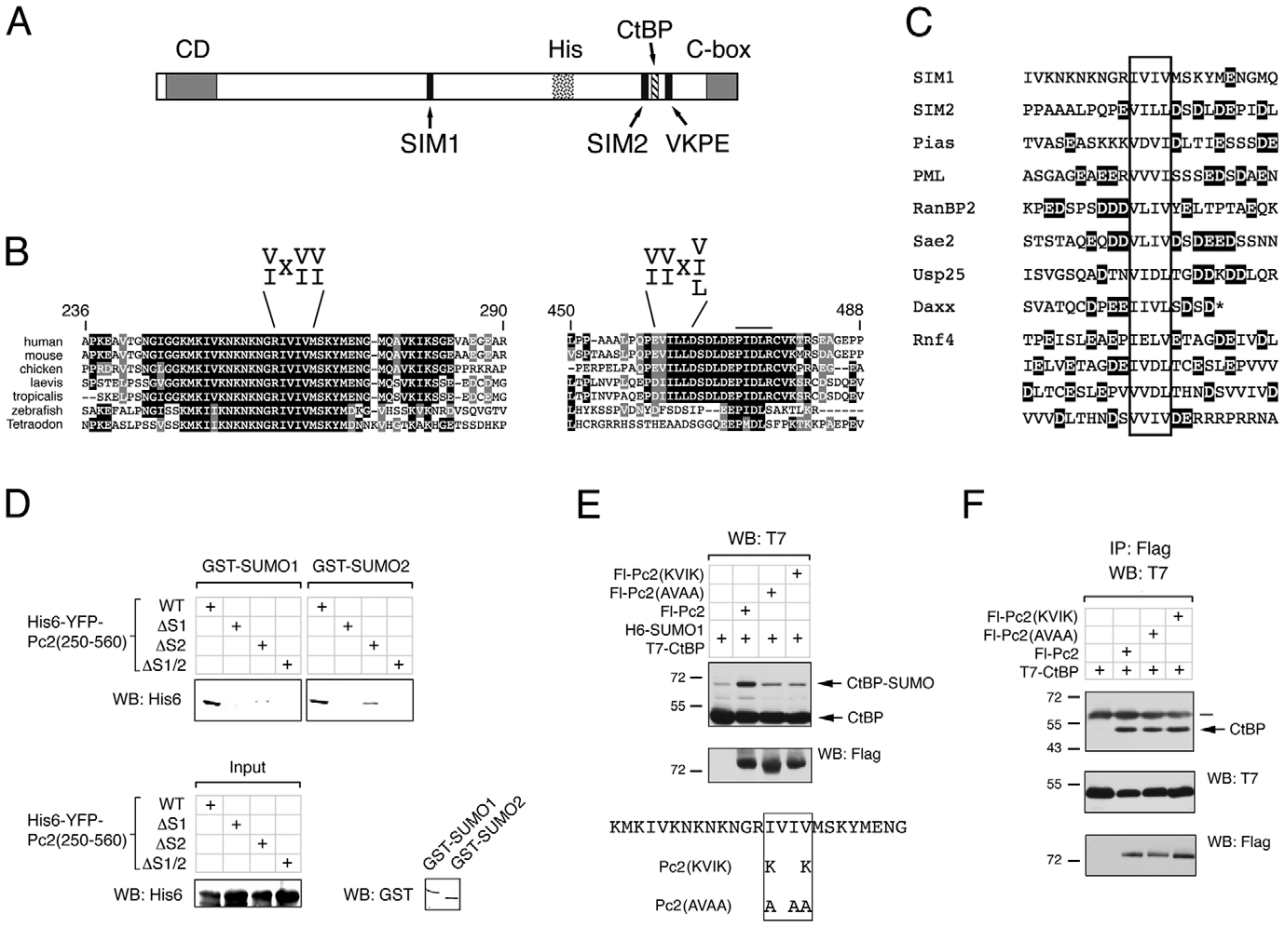
Pc2 contains two potential SIMs. A) Human Pc2 is shown schematically, with the location of the CtBP-interaction motif (PIDLR in Pc2), the major sumoylation site in Pc2 (VKPE) and the two consensus SIMs. CD: chromodomain, His: poly-histidine stretch, C-box: carboxyl-terminal homology box. B) Alignments of the conserved regions surrounding SIM1 and SIM2 are shown, together with the SIM consensus sequences. Amino acid number are from human Pc2, sequences are from human, mouse, chicken, Xenopus laevis and tropicalis, zebra fish and Tetraodon nigroviridis. The PIDLR, adjacent to SIM2, is indicated by a line above the sequence. Identity (black) and similarity (gray) in at least 5/7 sequences is shown. C) An alignment of known SIMs (protein names to the left) is shown. The SIM in each is boxed, and acidic residues within 10 amino-acids of the SIM are shaded. D) His6-YFP fusion proteins encoding amino acids 250–560 of Pc2 (either wild type, or lacking SIM1, SIM2 or both, as indicated) were incubated with GST fusions to SUMO1 or SUMO2 bound to glutathione agarose, and interacting proteins were identified by western blot. A portion of the input proteins was analyzed by western blot (below), with either a His6 antibody for the Pc2 fusions, or a GST antibody for the SUMO fusions. E) COS1 cells were transfected with the indicated expression constructs and lysates analyzed for CtBP and sumoylated CtBP. F) The interaction of wild type or SIM1 mutant Pc2 with CtBP was determined by co-immunoprecipitation from COS1 cells transfected as indicated. Proteins were precipitated on anti-Flag agarose and analyzed by T7 western blot. Expression in the lysates was monitored by direct western (lower panels). The sequence around SIM1 and of the two Pc2 SIM1 mutants are shown below. The positions of molecular weight markers are indicated.

Inspection of the amino acid sequence of Pc2 reveals the presence of two conserved SIMs (Figure 1A, B). One of these (SIM2) is in the carboxyl-terminal region of Pc2, very close to the CtBP interaction motif and the primary sumoylation site on Pc2. Comparison of this region of Pc2 with homologs from a number of vertebrate species reveals that it is conserved in mammals, birds and frogs, but is not present in Pc2 homologs from two fishes. In contrast, the adjacent CtBP binding motif (PxDL[R/S/T]) is present in all of the species shown in Figure 1. The more amino terminal SIM (SIM1) is again in a region of relatively high sequence conservation (Figure 1 and [39]), and is present in all vertebrate species examined. These two SIMs consist of a hydrophobic core, with the more carboxyl terminal one in the reverse orientation, such that position 3 is a non-hydrophobic residue. Examination of the sequences surrounding the two Pc2 SIMs reveals that SIM2 has several acidic residues in close proximity to it, as well as a conserved serine that is within a consensus CK2 phosphorylation site. SIMs in several other proteins share this property of being surrounded by acidic and potentially phosphorylatable residues (Figure 1C). Interestingly, the more amino terminal SIM1 is in a very highly conserved block of sequence, but this almost completely lacks acidic residues (Figure 1C). To confirm that the consensus SIMs in Pc2 could indeed mediate SUMO binding, we incubated bacterially expressed fusion proteins encoding amino acids 250–560 of Pc2 with GST fusions to SUMO1 or SUMO2, which had been purified form bacteria and immobilized on glutathione agarose. As shown in Figure 1D, the wild type Pc2 fusion bound to both SUMO1 and SUMO2, whereas deletion of either SIM1 or SIM2 alone (or both together) clearly decreased the interaction with both SUMO1 and SUMO2. Thus, it appears that Pc2 is capable of interacting directly with SUMO1 and SUMO2, and that the consensus SIMs in Pc2 are required for this interaction.

Based on the apparent difference from other SIMs, and the fact that SIM1 is in a region of Pc2 which we have previously shown to have E3 activity, we decided to analyze this SIM functionally. As a first test of whether SIM1 is important for Pc2 E3 function, we created two mutant forms of Pc2 in which two lysines or three alanines had been introduced into the IVIV of SIM1 (Figure 1E). COS1 cells were cotransfected with T7-tagged CtBP and six histidine-tagged SUMO1 (H6-SUMO1), together with wild type or mutant Pc2, or a control vector. Cells were then lysed and subjected to direct western blotting for T7-CtBP and the sumoylated form of the protein. As shown in Figure 1E, coexpression of wild type Pc2 increased the amount of sumoylated CtBP compared to cells transfected with CtBP and SUMO1 alone, whereas, this increase was not seen with either of the mutant Pc2 constructs. To verify that these two mutants were otherwise functional, we coexpressed them together with T7-CtBP and precipitated proteins on anti-Flag agarose. T7-CtBP coprecipitated to a similar degree with Flag-tagged wild type Pc2 and each of the mutants, whereas no CtBP was visible in a control precipitate (Figure 1F). These data suggest that the Pc2 SIM1 is important for Pc2 E3 activity *in vivo*.

### Identification of a Minimal Functional Domain Surrounding SIM1

**Figure 2:**
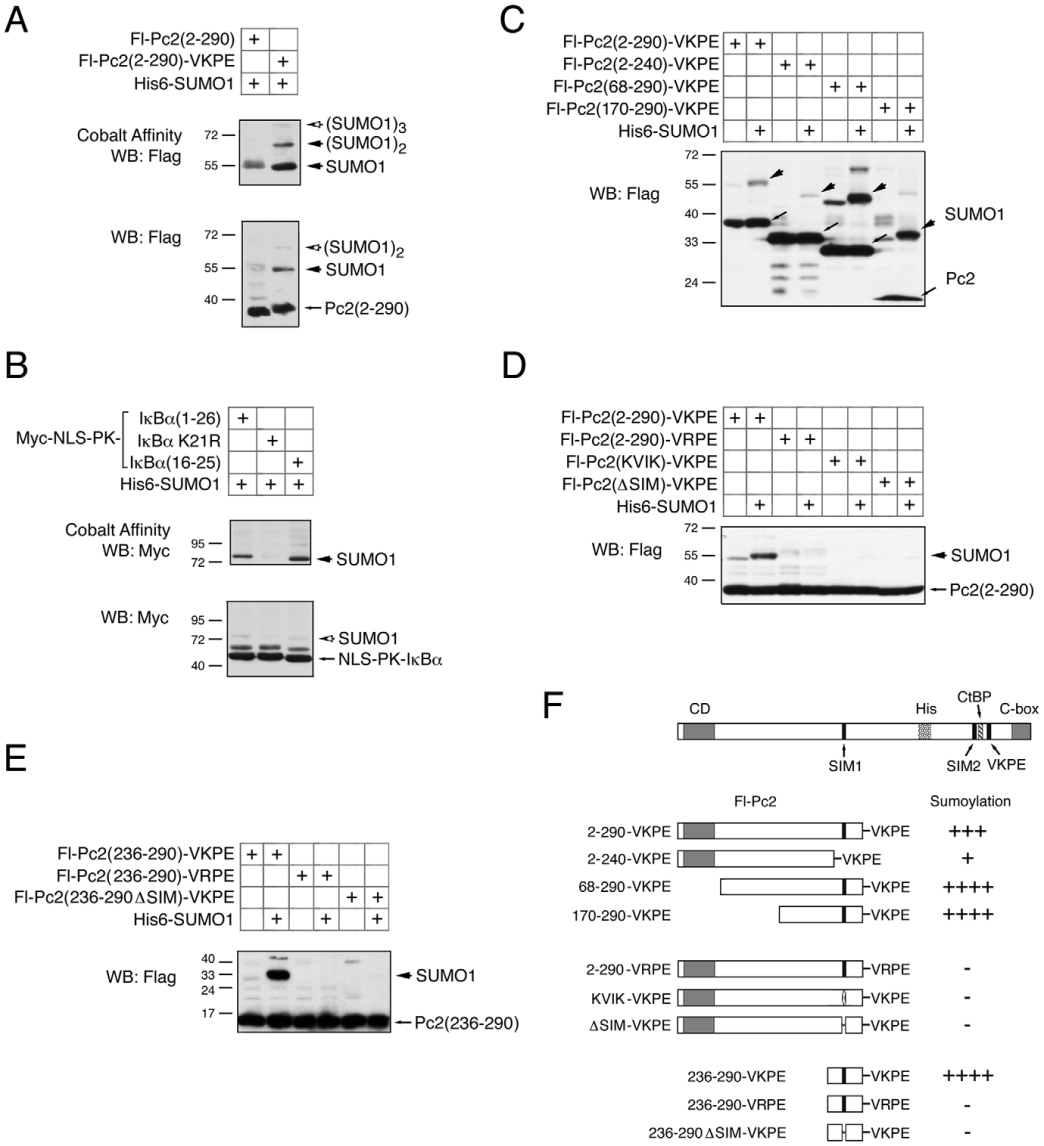
Identification of a minimal functional domain around SIM1. A) COS1 cells were transfected with Flag-tagged Pc2(2-290), or a version with the VKPE sequence added after amino acid 290, and analyzed by cobalt affinity and Flag western blot (above) or by direct western on the lysates (below). The positions of the unmodified Pc2 and the sumoylated forms are shown). B) Cells were analyzed as in A, using a series of fusions of the NLS and sumoylation site from IκBα, with Myc epitope-tagged pyruvate kinase. C) A series of Pc2 deletion mutants was expressed in COS1 cells with or without co-expressed His-tagged SUMO1, and analyzed by direct Flag western blot, as in A. For each pair of lanes, the Pc2-VKPE fusion is indicated with an arrow, and the major sumoylated with an arrowhead. In lanes 1, 5 and 7, modification with endogenous SUMO is also detected. D and E) Lysates from transfected COS1 cells were analyzed as in C, for a series of Pc2(2-290)-based mutants (D), or Pc2(236-290)-based mutants (E), as indicated. F) The Pc2 constructs used in this figure are shown schematically, together with a summary of their sumoylation status in the presence of co-expressed SUMO1. The positions of molecular weight markers are indicated.

To simplify analysis of SIM1, we created a fusion protein in which the amino-terminal 290 amino acids of Pc2 were fused to a carboxyl-terminal consensus sumoylation motif (VKPE), followed by a stop codon, and an amino-terminal Flag epitope tag (see Figure 2F). To verify that this construct [(2-290)-VKPE] could indeed be sumoylated in cells, we coexpressed it in COS1 cells with H6-SUMO1. Cells were then lysed in 6M guanidine HCl, and histidine tagged proteins isolated on cobalt agarose. As shown in Figure 2A, when the cobalt bound fraction was western blotted with a Flag antibody sumoylated forms of the VKPE fusion were clearly visible, but were not seen with a similar construct lacking the VKPE peptide. It has previously been shown that addition of a sumoylation consensus and a nuclear localization sequence (NLS) is enough to drive some sumoylation of a reporter protein [45]. We therefore compared the level of sumoylation of such constructs to Pc2(2-290)-VKPE, to begin to test whether its sumoylation was due to more than simply driving nuclear localization of a sumoylation consensus site. As shown in Figure 2B, sumoylation of Myc-epitope tagged pyruvate kinase fused to two peptides from IκBα was readily detected by cobalt precipitation and Myc western blot. However, the sumoylated form of this reporter protein represented only a very small fraction of the total protein, when analyzed by direct western blotting of cell lysates. In contrast sumoylated Pc2(2-290)-VKPE constituted a much larger proportion of the total (compare the lower panels in Figures 2A and B). Thus the Pc2(2-290)-VKPE fusion is becoming sumoylated to a greater degree than would be expected for a protein that simply contains an NLS and a sumoylation consensus site, suggesting the presence of an additional activity within this fusion protein. We next tested a series of deletion constructs within the context of the VKPE fusion. A carboxyl-terminal truncation to amino acid 240, which removes SIM1, greatly reduced modification of the VKPE fusion (Figure 2C). In contrast, amino-terminal deletions either to amino acid 68 or 170 actually increased the proportion of sumoylated protein, and modification by endogenous SUMO was readily apparent. To test the importance of SIM1 in this context, we analyzed two versions of the (2-290)-VKPE fusion, in which the SIM1 had either been mutated to KVIK or deleted. As shown in Figure 2D, both of these mutations essentially abolished modification of this construct. To verify that the sumoylation we were seeing was occurring within the VKPE motif, we also tested a wild type fusion to a VRPE peptide. No sumoylation of this construct [(2-290)-VRPE] was observed (Figure 2D). To test whether the region of sequence conservation surrounding SIM1 was sufficient to promote sumoylation we tested a construct which contained only 55 amino acids from Pc2 [(236-290)-VKPE]. As shown in Figure 2E, this construct was robustly sumoylated, whereas a version in which the SIM had been deleted, or a fusion to a VRPE peptide were unmodified. Together, these data suggest that the conserved region surrounding SIM1 is sufficient to promote modification of a linked consensus sumoylation site, and that the SIM is essential for this activity.

### Mutational Analysis Around SIM1

**Figure 3:**
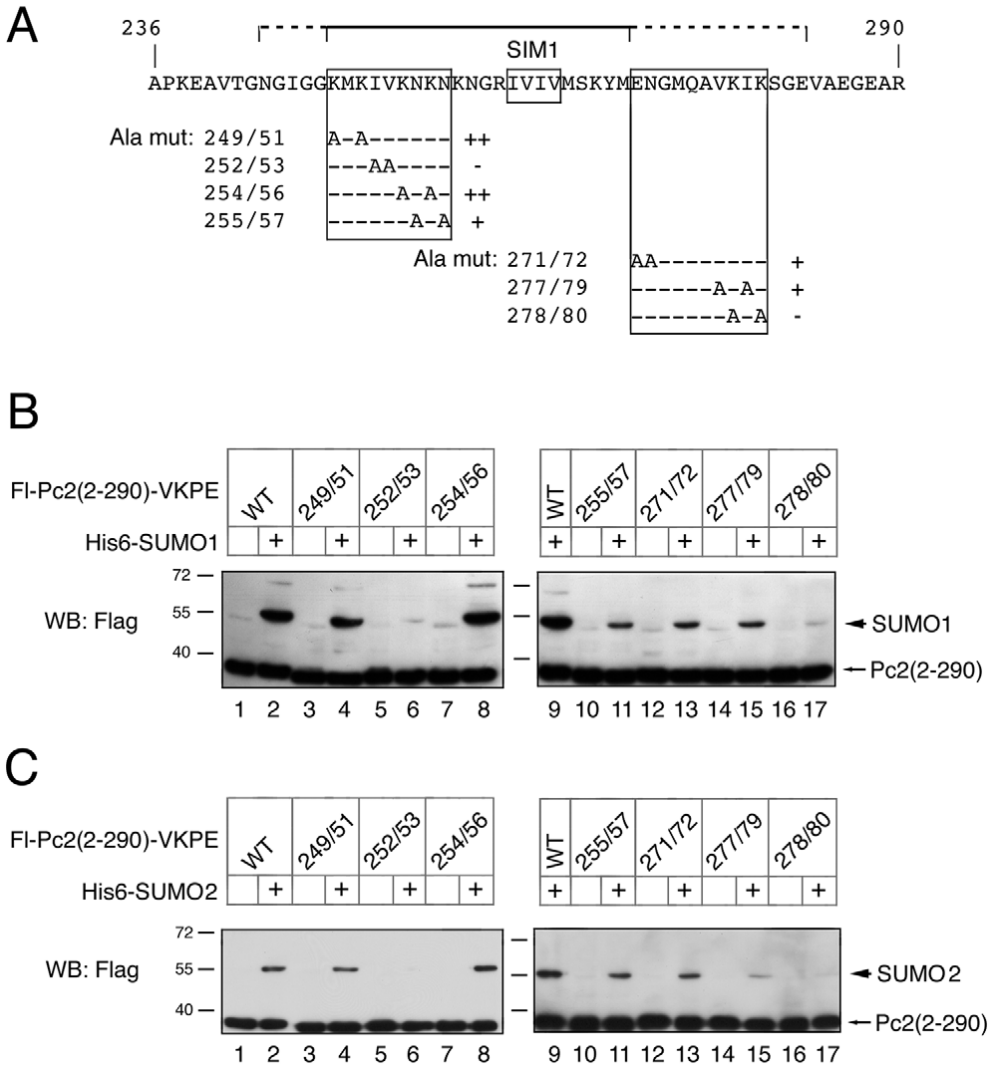
Mutational analysis of the SIM1 surroundings. A) The minimal sequence surrounding SIM1 shown to have E3 activity is shown. The line and dashed lines above represent the regions of highest similarity across species (see Figure 1). The alanine mutants analyzed are shown below, together with a summary of their activity. B and C) COS1 cells were transfected with a series of Flag-Pc2(2-290)-VKPE expression constructs, in which pairs of conserved residues surrounding the SIM were altered to alanines. Constructs were expressed with or without co-expressed His-tagged SUMO1 (B) or SUMO2 (C). Lysates were analyzed by direct Flag western blot to detect unmodified and sumoylated Pc2 constructs. The positions of molecular weight markers are indicated.

The core of the 55 amino acids surrounding SIM1 are highly conserved across diverse vertebrate species. To identify other amino acids which might be important for SUMO binding, we performed alanine scanning mutagenesis across this region, within the context of the Flag-Pc2(2-290)-VKPE fusion protein. Eight double alanine mutants were tested, in which we primarily focused on charged and hydrophobic residues. Seven of the eight were expressed at similar levels to the wild type protein, and are shown in Figure 3A. We next tested each of these seven mutants for modification by SUMO1. As shown in Figure 3B, alteration of lysines 278 and 280 to alanine effectively abolished the activity of this construct, whereas mutation of lysines amino terminal to the SIM had little or no effect (compare lane 17 with lanes 2, 4 and 8). Mutation of two amino-terminal hydrophobic residues (IV 252/253) also completely abolished activity, whereas the VI 277/279 mutation had a more minimal effect (lanes 6 and 15). Additionally, mutation of two asparagines (255/257) as well as the EN 271/272 mutation somewhat reduced activity (Figure 3B, lanes 11 and 13). A similar set of analyses was also performed using SUMO2 in place of SUMO1, with similar results (Figure 3C). This analysis demonstrates that several conserved amino acids surrounding the SIM1 are essential for promoting sumoylation in the context of these fusion proteins, and suggests that they may play a role in SUMO binding.

**Figure 4:**
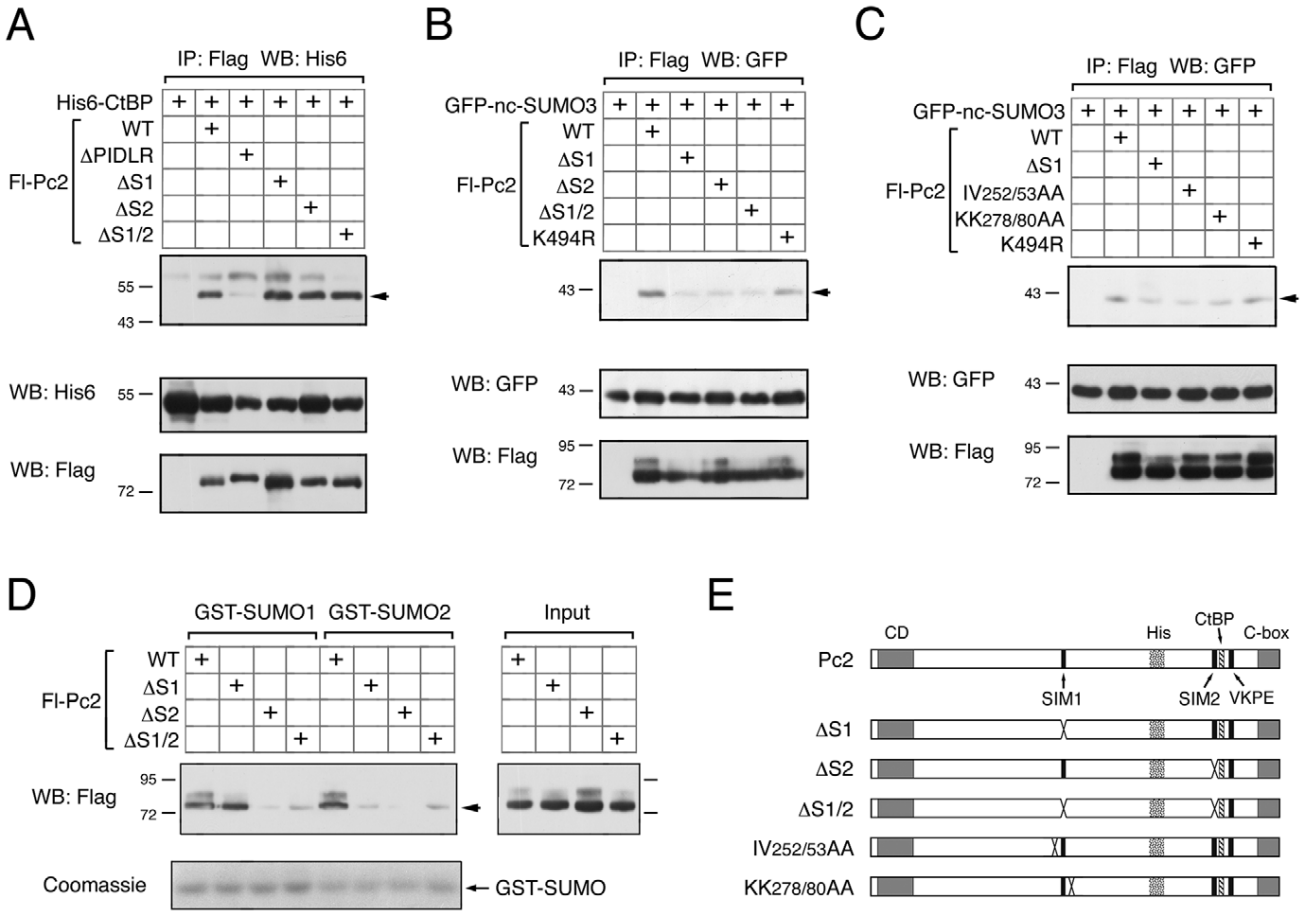
Pc2 SIMs are required for interaction with SUMO3. A) A series of Pc2 expression constructs with either SIM1, SIM2 or both deleted (ΔS1, ΔS2, ΔS1/2, respectively) was created, and tested for interaction with CtBP by coimmunoprecipitation from transfected COS1 cells, as in Figure 1. The CtBP interaction mutant (ΔPIDLR) was included as a control. Co-precipitating CtBP is indicated by an arrow, the upper band is the heavy chain. Expression in the lysates is shown in the lower panels. B and C) COS1 cells were transfected with the indicated Flag-Pc2 expression constructs together with a vector encoding a GFP-tagged non-conjugatable mutant of SUMO3 (GFP-nc-SUMO3). Proteins were precipitated on anti-Flag agarose and analyzed by western blot with a GFP antibody. Expression in the lysates is shown below. Arrows indicate co-precipitating proteins. D) Lysates from COS1 cells transfected with the indicated Pc2 constructs were incubated with glutathione agarose to which recombinant bacterially expressed GST-SUMO1 or GST-SUMO2 had been pre-bound. Bound proteins were analyzed by western blot for Flag-Pc2 constructs. A portion of the lysate was analyzed in parallel (input), and the GST-SUMO fusions were visualized by Coomassie blue staining the lower part of the gel. E) The Pc2 expression constructs analyzed are shown schematically. The positions of molecular weight markers are indicated.

To begin to compare the importance of the two SIMs in Pc2 for E3 activity, we created a series of expression constructs in which the IVIV of SIM1 and the VILL of SIM2 had been deleted (see Figure 4E). Single and double SIM deletions were generated in the context of full length Pc2, or a truncated form (encoding amino-acids 2-531) that lacks the carboxyl-terminal 29 amino acids (the C-box), and is delocalized from polycomb bodies [38], [40]. Additionally, we transferred the two most severe alanine mutations (IV 252/253 and KK 278/280) to both full length Pc2, and the Pc2 deletion lacking the C-box. We first verified that SIM deletions did not affect interaction with CtBP by coimmunoprecipitation from transfected COS1 cells. As shown in Figure 4A, SIM deletions either alone or in combination had no affect on CtBP interaction, whereas deletion of the PIDLR CtBP interaction motif significantly weakened CtBP binding. We next tested the effect of deleting the SIMs on interaction with a non-conjugatable version of SUMO3 (GFP-nc-SUMO3) in transfected COS1 cells. Flag-tagged Pc2 proteins were collected on anti-Flag agarose, and probed for the presence of co-precipitating GFP-nc-SUMO3. As shown in Figure 4B, deletion of either SIM1 or SIM2 reduced the interaction of Pc2 with nc-SUMO3. In contrast, alteration of the primary sumoylated lysine in Pc2 to arginine (K494R) had little effect. Similar experiments using the IV 252/253 and KK 278/280 mutant forms of full length Pc2 also revealed a decrease in the interaction of these Pc2 mutants with nc-SUMO3 (Figure 4C). To further compare SUMO binding by SIM mutant forms of Pc2 we tested interaction of GST-SUMO1 or GST-SUMO2, purified from bacteria, with Flag-tagged Pc2 expressed in COS1 cells. As shown in Figure 4D, Pc2 and the SIM1 deletion mutant bound to GST-SUMO1, whereas binding of the SIM2 or double SIM mutant was clearly reduced. As with the results from co-transfection of Pc2 with nc-SUMO3, both single SIM mutants reduced binding to GST-SUMO2. Together, this data suggests that both SIMs in Pc2 can contribute to non-covalent SUMO binding, but that SIM1 plays less of a role with SUMO1.

### An *In Vivo* Role for SIM1 and SIM2 in Pc2 E3 Activity

**Figure 5:**
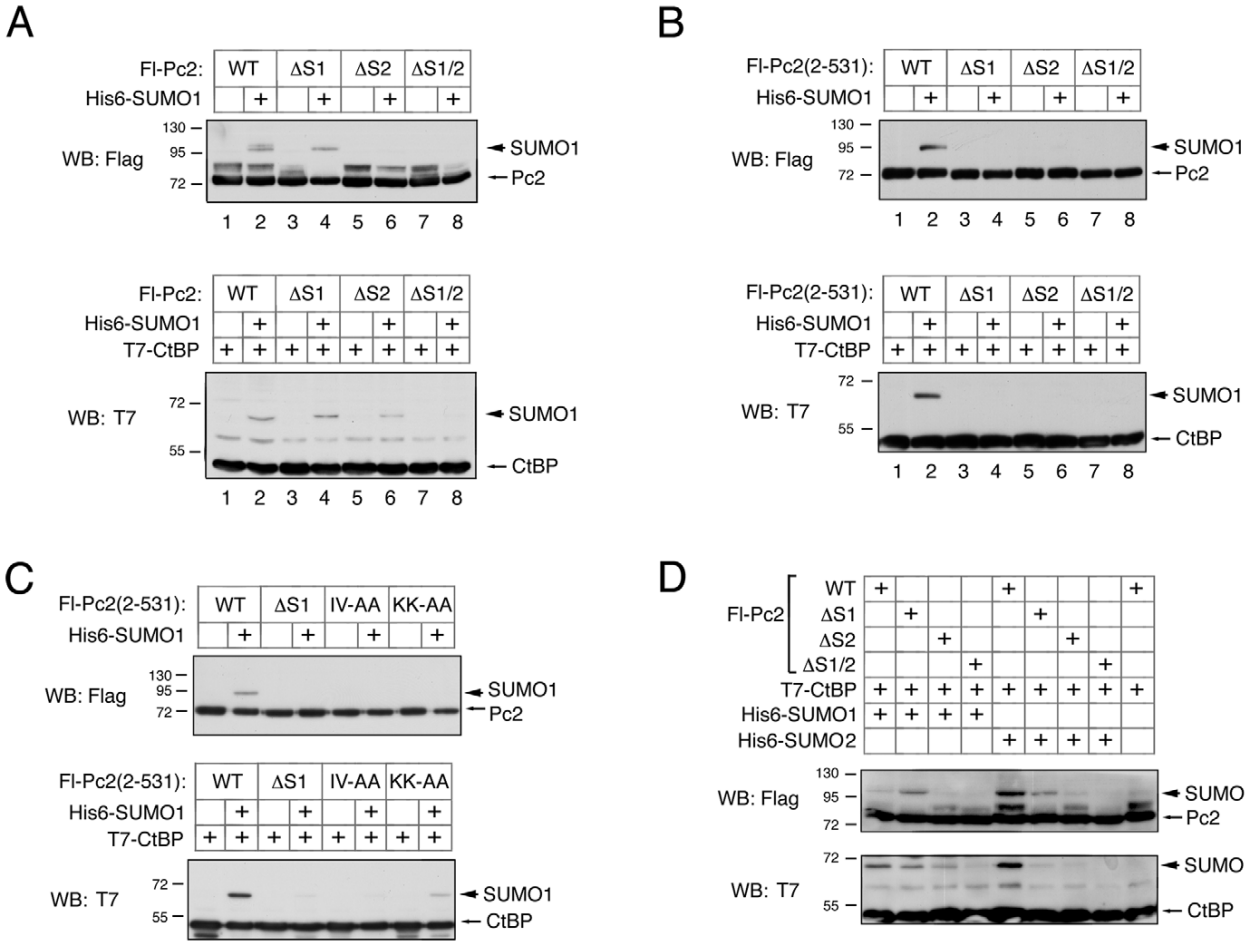
SIM1 and SIM2 contribute to E3 activity. A) Sumoylation of Pc2 and Pc2 mutants (upper panel) was tested by direct western blot in lysates from transfected COS1 cell either without or with coexpressed His6-SUMO1. Lower panel shows sumoylation of CtBP, analyzed by direct western blot of COS1 cell lysates, transfected with the series of Pc2 constructs. B) Sumoylation of Pc2 (above) and CtBP (below) was analyzed as in A, except that all Pc2 expression constructs are all in the context of the 2-531 deletion mutant of Pc2, which lacks the carboxyl-terminal domain (C-box) and is delocalized from polycomb foci. C) Sumoylation of Pc2 (upper panel) and CtBP (lower panel) was analyzed by direct western blot as in A and B. Only the series using Pc2 and mutants in the context of amino-acids 2-531 is shown, as these mutations did not affect the full length protein. D) Sumoylation of Pc2 (upper panel) and CtBP (lower panel) in the presence of either coexpressed SUMO1 or SUMO2, as indicated, was analyzed by direct western blot. Wild type Pc2 and SIM deletion mutants in the context of full length Pc2 are shown. The positions of molecular weight markers are indicated.

We next analyzed the effects of SIM mutations on SUMO modification of Pc2 and CtBP. Each of the Pc2 SIM deletion constructs was coexpressed in COS1 cells with SUMO1, and analyzed by direct western blotting for the Flag tag on Pc2. Mutation of SIM1 in the context of full length Pc2 had little effect on Pc2 sumoylation, but completely abolished it in the context of the delocalized Pc2(2-531) (Figure 5A and B, compare lanes 2, 4 in each panel). In contrast, mutation of SIM2 alone or in combination with SIM1 completely abolished Pc2 sumoylation in either context (Figure 5A, B). To test modification of a recruited substrate protein, we performed a similar set of analyses in the presence of coexpressed CtBP. As shown in Figure 5A, mutation of SIM2 alone reduced CtBP sumoylation, whereas deletion of SIM1 had little effect. However, deletion of both SIMs in combination, greatly reduced E3 activity towards CtBP. In the context of the delocalized Pc2(2-531) truncation mutant, deletion of either SIM alone abolished E3 activity towards CtBP (Figure 5B). Thus it appears that both SIMs contribute to full E3 activity towards CtBP, whereas there is a lesser requirement for SIM1 for auto-sumoylation of Pc2. Analysis of the two alanine mutants (IV 252/253 and KK 278/280) in the context of full length Pc2 revealed no discernible effect on sumoylation of either Pc2 or CtBP (data not shown). However, in the context of the de-localized Pc2(2-531) construct, modification by SUMO1 of both Pc2 and CtBP was reduced by these point mutations (Figure 5C). Since the alanine mutations surrounding SIM1 appeared to affect modification by both SUMO1 and SUMO2, in the context of the amino-terminal Pc2 fusions, we next compared modification of CtBP and full length Pc2 by SUMO1 and SUMO2 in parallel. COS1 cells were transfected with CtBP and Pc2 or one of the SIM deletion mutants, together with either SUMO1 or SUMO2. As shown in Figure 5D, deletion of SIM1 did not reduce sumoylation of Pc2 and in this assay only slightly reduced CtBP modification by SUMO1, whereas deletion of SIM2 alone or in combination with SIM1 dramatically reduced SUMO1 modification of both proteins. Interestingly, with SUMO2 deletion of SIM1 alone clearly reduced modification of both Pc2 and CtBP. Together, these data suggest that SIM2 is required for Pc2 E3 activity with either SUMO1 or SUMO2, whereas, in the context of full length Pc2, SIM1 appears to contribute primarily to E3 activity with SUMO2.

### SIM Deletions Delocalize SUMO from Pc2 Foci

**Figure 6:**
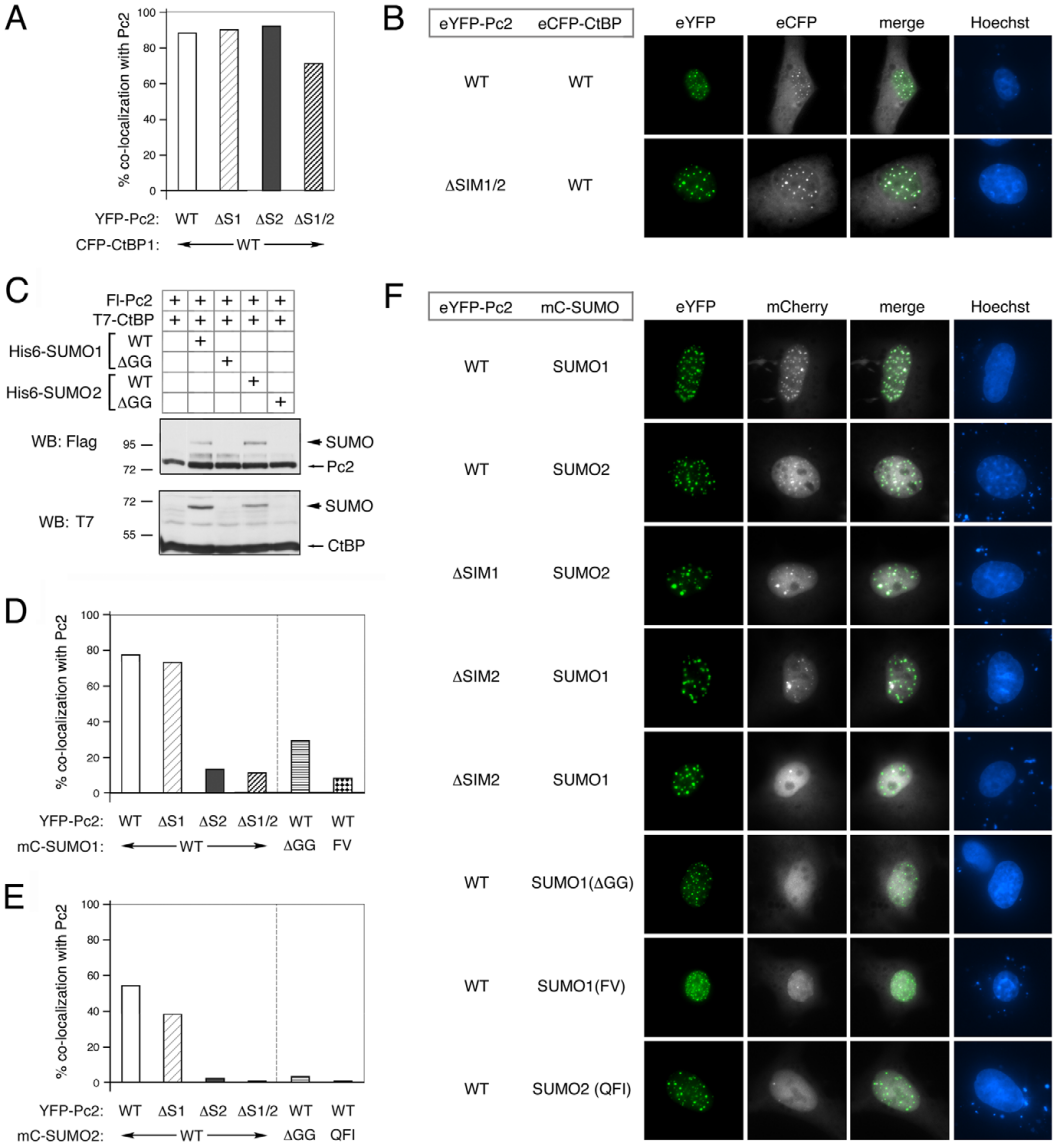
Sub-cellular localization of SUMO and Pc2 SIM mutants. A) COS1 cells were transfected with the indicated eYFP-tagged Pc2 expression constructs with eCFP-tagged CtBP and visualized by live cell fluorescence microscopy. Cells with Pc2 foci were identified and scored for colocalization of eCFP-CtBP. The percentage of cells with colocalization is plotted for each Pc2 mutant. B) Examples of CtBP and Pc2 colocalization are shown. Separate YFP and CFP images (false colored to green and white for maximal contrast) are shown, together with a merged image and the corresponding Hoechst stain. C) Modification of Pc2 and CtBP was analyzed by direct western blot of COS1 cell lysates transfected with the indicated wild type or non-conjugatable (ΔGG) versions of SUMO1 and SUMO2. The positions of molecular weight markers are indicated. D and E) The proportion of cells in which the indicated monomeric Cherry (mC-) SUMO1 (D) and SUMO2 (E) fusions co-localized with the indicated eYFP-Pc2 constructs is shown. The SUMO expression constructs are either wild type, ΔGG (as in panel C), or are the SUMO1(FV) and SUMO2(QFI) SIM interaction mutants shown schematically in Figure 7D. F) Representative live cell fluorescent images showing localization of a selection of Pc2 and SUMO expression constructs are shown. The coexpressed eYFP-Pc2 and mC-SUMO constructs are indicated to the left, and individual eYFP (in green) and mCherry (in white) images are shown, together with the merged image and the Hoechst stain.

Pc2 can recruit CtBP and SUMO1 or SUMO2 to sub-nuclear foci, termed polycomb bodies [18], [38]. This provides a useful assay to examine the requirements for assembly of the Pc2-containing sumoylation complex, and subsequent substrate sumoylation, in living cells. To test the effects of SIM mutations on the recruitment of SUMO1 and SUMO2, we created eYFP-tagged versions of the single and double SIM deletion mutants of Pc2. COS1 cells were transfected with individual eYFP-Pc2 constructs, imaged at 22 hours after transfection, and the distribution of Pc2 fluorescence scored as soon as a significant number of transfected cells were readily visible. By analyzing the cells only 22 hours post-transfection we hoped to avoid problems due to high level over-expression of the transfected proteins. The wild-type and each of the three mutant forms of Pc2 all formed sub-nuclear foci, in the majority of cells, although deletion of SIM1 resulted in an increase in the proportion of cells with foci that were larger than those seen with wild type Pc2 (Figure 6B and F, and data not shown). We did not observe an increase for any of the mutants in the proportion of cells with a diffuse nuclear pattern of Pc2 localization, as seen with the C-box deletion.

To test the ability of these mutants to recruit other proteins, we coexpressed the eYFP-tagged Pc2 constructs together with eCFP-tagged CtBP. Cells with Pc2 foci were then selected and the presence of eCFP foci scored, to determine what proportion of cells with typical Pc2 localization had co-localization of the interacting partner. Deletion of either SIM alone, or both together had minimal effect on CtBP recruitment to Pc2 foci, whether they were the large or small foci (Figure 6A and B). Thus it appears that all three SIM mutant forms of Pc2 localize normally and can recruit a partner protein. We next created fusions of SUMO1 and SUMO2 to the monomeric Cherry fluorescent protein (referred to here as mC-fusions). Additionally we created non-conjugatable mutants (ΔGG mutants) of SUMO1 and -2, in which the di-glycine motif that is required for removal of the carboxyl-terminal tail had been altered to two alanines. As shown in Figure 6C, the ΔGG mutants of both SUMO1 and SUMO2 were unable to be attached to either CtBP or Pc2 when expressed in COS1 cells. To test whether deletion of the SIMs in Pc2 affected localization of SUMO1 or SUMO2 to polycomb foci, we coexpressed wild type or SIM mutant eYFP-tagged Pc2 together with mC-SUMO fusions in COS1 cells. Cells were examined as for CtBP co-localization and scored for colocalization of the SUMO fusion protein with Pc2. mC-SUMO1 co-localized with Pc2 in almost 80% of cells with Pc2 foci, whereas for SUMO2, the degree of colocalization was less than 60% (Figure 6D and E). Examples of the different kinds of Pc2 and SUMO localization observed are shown in Figure 6F. For both SUMO1 and SUMO2, deletion of SIM1 had little effect on the proportion of cells with clear colocalization, although with SUMO2 it did drop to below 40% (Figure 6E). Deletion of either SIM2 alone, or in combination with SIM1 dramatically reduced colocalization of either SUMO1 or SUMO2, consistent with the more important role for SIM2 in E3 activity. We next tested whether the ΔGG SUMO mutants were able to colocalize with Pc2. For SUMO1, the proportion of cells in which we observed colocalization decreased to less than 30% with the ΔGG mutant, and for SUMO2 colocalization was effectively abolished (Figure 6D and E). Together, these data suggest that the observed localization of SUMO to Pc2 foci is SIM dependent, and requires the SUMO to be competent for processing and substrate modification.

### SIM Interaction Mutant SUMOs Are Not Competent for Pc2 E3 Activity

**Figure 7:**
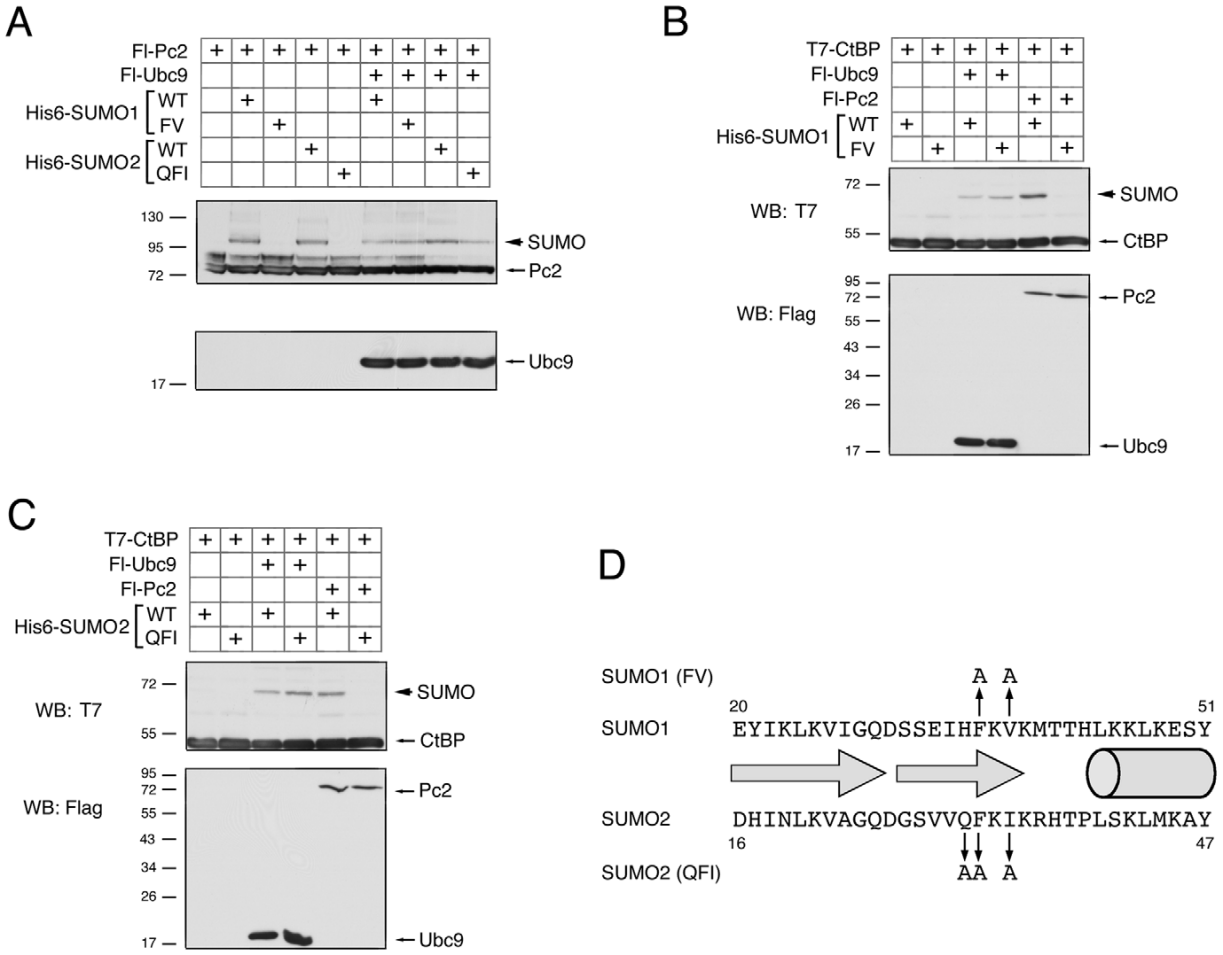
Mutational analysis of SUMO1 and SUMO2. A) Modification of wild type Flag-tagged Pc2 by the indicated His6-tagged SUMO variants in transfected COS1 cells was analyzed by direct western blot. B and C) Modification of CtBP by the indicated SUMO variants alone or coexpressed with Ubc9 or Pc2 was analyzed as in A. Expression of the Flag-tagged Pc2 and Ubc9 is shown in the lower panels. D) Partial amino acid sequences of SUMO1 and SUMO2 are shown. Numbers indicate amino acid numbers. The two arrows indicate the positions of β strands 1 and 2, and the cylinder the first α helix. The residues in each SUMO isoform which were altered to alanines to create the FV and QFI mutants are shown. The positions of molecular weight markers are indicated.

The residues within SUMO, which contribute to non-covalent interaction with SIMs, have been identified [22], [23], [24]. Recent work demonstrated that for certain substrates, the presence of a SIM was required for efficient modification, and that mutation of amino acids 30, 31 and 33 of SUMO2 to alanines abolished SIM-dependent modification [29]. To test the requirement of SUMO-SIM interaction for Pc2 E3 activity, we generated a similar triple alanine mutant form of SUMO2 (QFI mutant, see Figure 7D), as well as a double point mutant form of SUMO1, in which phenyl alanine 36 and valine 38 of SUMO1 were converted to alanines (SUMO1 FV mutant, see Figure 7D). Each of these mutants was created in the context of a processed SUMO, which ended with the di-glycine motif at its carboxyl-terminus, and either a six-histidine tag or monomeric Cherry fusion at the amino-terminus. Colocalization of the mC fusions of these SUMO mutants with eYFP-Pc2 was analyzed as before. Coexpression of mC-SUMO2(QFI) with eYFP-Pc2 resulted in essentially no colocalization of the mutant SUMO2 with Pc2 (Figure 6E and F). For the SUMO1(FV) mutant, colocalization was observed in less than 10% of cells, below the level seen with the double SIM mutant Pc2 and wild type SUMO1 (Figure 6D and F). We next analyzed the FV and QFI mutants for their ability to be covalently attached to both Pc2 and CtBP in transfected COS1 cells. As shown in Figure 7A, both the SUMO1(FV) and SUMO2(QFI) mutants were severely impaired in their ability to be attached to Pc2. However, when we also over-expressed Ubc9 in these cells this was able to drive the modification of Pc2 by both of the SUMO mutants, to a level similar to that seen with the wild type proteins, suggesting that these SUMO mutants are functional for substrate modification (Figure 7A). We also compared modification of CtBP by wild type and SIM-interaction mutant SUMOs in the presence of co-expressed Ubc9 and Pc2. When SUMO1 or the FV mutant form were co-expressed with Ubc9, similar levels of CtBP modification were observed (Figure 7B). In the presence of co-expressed Pc2, SUMO1 modification of CtBP was clearly visible, whereas with the FV mutant no modified CtBP was observed. Similar results were obtained with SUMO2 and the QFI mutant, suggesting that both SUMO mutants can be conjugated to CtBP, but are not functional for Pc2 E3 activity (Figure 7C). Together, these data suggest that the SIM interacting residues in both SUMO1 and SUMO2 are required for Pc2 E3 activity *in vivo*, and support the idea that SIMs in Pc2 contribute to E3 activity.

## Discussion

Here we show that Pc2 contains two non-covalent SUMO-interaction motifs that are required for *in vivo* SUMO E3 activity. Consistent with this we show the SIM-interacting domains of both SUMO1 and SUMO2 are required for Pc2-dependent sumoylation of CtBP, but are dispensable when sumoylation is driven by high levels of Ubc9. This provides the first *in vivo* evidence for a role for non-covalent SIMs in SUMO E3 activity.

SUMO interaction motifs generally consist of a short hydrophobic core sequence, which often has acidic residues surrounding it [20], [21], [22], [23]. In addition, recent evidence has suggested that phosphorylation of CK2 sites adjacent to a SIM can increase the efficiency of SUMO binding [26]. The best defined SIMs consist of a hydrophobic block of four residues, where position 2 or 3 can be variable [22], [23]. Both SIMs identified in Pc2 conform to this consensus, having a stretch of four hydrophobics with no interruption. One of these, SIM2, has several acidic residues in the surrounding sequence, as well as a consensus CK2 phosphorylation site, although the importance of this remains to be tested. In contrast to SIM2, of the 20 amino acids surrounding SIM1 only one is acidic. Our mutational analyses have identified two pairs of residues surrounding SIM1 that contribute to SUMO interaction and Pc2 E3 activity, although it should be noted that mutating either pair alone has a more subtle effect on E3 activity than mutating or deleting the hydrophobic core. In the context of the isolated amino-terminal half of Pc2, mutation of either of these pairs of amino acids, one of which consists of two hydrophobic residues, the other two basic residues, had more effect on E3 activity than mutating the single acidic residue within the proximity of SIM1. No other convincing candidate SIMs are present in Pc2, although SIM consensus sequences can be somewhat variable. In Pc2 other small patches of hydrophobic residues are present, including three isoleucines close to the extreme carboxyl-terminus of the protein, which have been suggested to be important for E3 activity (A.D.S. and S.H.Y., unpublished). Additionally, it is possible that SUMO might form non-covalent interactions via other binding surfaces. Much of our analysis of the effect of Pc2 SIM mutations has relied on CtBP sumoylation as a readout for Pc2 E3 activity. Analysis of the sequence of CtBP reveals several groups of hydrophobic amino acids that resemble the SIM core consensus. However, examination of the structure of CtBP suggests that none of these regions are surface exposed, such that they are unlikely to be able to make contact with SUMO. However, for some SUMO substrates, the presence of a hydrophobic SIM can drive sumoylation, and it has been suggested that the SIM acts to recruit SUMO-loaded Ubc9 to the substrate [29]. In our assays SIM-interaction mutants of both SUMO1 and SUMO2 are competent for modification of CtBP when driven by over-expression of Ubc9, suggesting that in the absence of Pc2 SIM-SUMO interactions do not contribute to CtBP modification.

Among the first proteins to be shown to have hydrophobic SIMs were members of the PIAS family and RanBP2/Nup358 [22]. This clearly raised the possibility that SIMs in components of the sumoylation machinery may contribute to sumoylation, and in the case of PIAS and RanBP2/Nup358 might be required for full E3 activity. However, the minimal domain of RanBP2/Nup358 required for *in vitro* E3 activity does not include the SIM, although it is directly adjacent to it, and it has been shown to bind SUMO *in vitro* [22], [29]. For the PIAS family the case for a role of SIMs in E3 activity is even less clear. Conserved SIMs are found in all members of the mammalian PIAS family, but we (J.C.M., M.H.K. and D.W., unpublished) and others have shown that the SIM in PIAS1 is not required for E3 activity [26]. Intriguingly the SIM in PIAS1 has been shown to contribute to its activity as a transcriptional coregulator. Thus the PIAS1 SIM may be functioning more like the SIM in CoREST1, to recruit transcriptional regulators that are themselves targets for covalent SUMO modification [25], [26]. We have not analyzed the effect of Pc2 SIM mutations on transcriptional regulation by Pc2 since they had clear effects on E3 activity. However, if E3 activity is required for transcriptional regulation by Pc2, such mutations would be expected to affect Pc2 mediated repression. Alternatively, recruitment of sumoylated proteins to Pc2 containing PRC complexes might be in part dependent on SUMO interactions with the Pc2 SIMs.

This work clearly demonstrates that non-covalent SUMO binding by Pc2 is required for E3 activity *in vivo*, raising the possibility that SIMs in other components of the sumoylation pathway may play a role. The fact that the SIM-interaction mutations of both SUMO1 and SUMO2 result in similar defects in Pc2-dependent CtBP modification, but do not completely inactivate SUMO provides further support for the idea that the E3 activity of Pc2 requires non-covalent interactions with SUMO. One possibility, is that one or both of the Pc2 SIMs play a role in recruiting SUMO-loaded Ubc9. Thus active Ubc9 with SUMO attached to the catalytic cysteine would bind preferentially to Pc2 and once the SUMO had been transferred to substrate, this interaction would be weakened, allowing for exchange of the unloaded for loaded Ubc9. So far we have not been able to show any effect of the SIM mutations in Pc2 on Ubc9 recruitment, but this remains an attractive model. Recent evidence has suggested that Ubc9 itself can be covalently modified with SUMO on lysine 14, and that this allows for preferential recruitment of sumoylated Ubc9 to some substrates [46]. This would also provide a potential mechanism for the role of SIMs in Pc2 activity – sumoylated (on K14) Ubc9 might be preferentially recruited in part via SUMO-SIM interactions, thereby promoting the transfer of the loaded SUMO from the catalytic cysteine to substrates such as CtBP. However, we have been unable to show any effect of mutating lysine 14 in Ubc9 on Pc2 modification, E3 activity or on Ubc9 recruitment (data not shown). An alternative possible explanation for the importance of SIM-SUMO interactions in Pc2 E3 activity is that these interactions do not promote recruitment of Ubc9, but that the interaction of the SIM with SUMO promotes the transfer of SUMO from Ubc9 to substrate by positioning the SUMO appropriately. Our data suggest that SIM2 is the more important of the two SIMs in Pc2, although we have not been able to clearly show that one SIM performs a function that the other does not. However, it is possible that one SIM might contribute preferentially or even exclusively to modification of some Pc2 E3 substrates. In this context, SIM2 does appear to play a larger role in sumoylation of both CtBP and Pc2 itself than does SIM1, although there is clearly a role for SIM1. An additional possibility is that at physiological levels of expression SIM1 might promote only the transfer of SUMO2 to substrates, since it appears less able to work with SUMO1, even when over-expressed. This is consistent with data comparing binding of multiple different SIMs to both SUMO1 and SUMO2, which suggests that negatively charged amino acids surrounding the hydrophobic core contribute to SUMO1 binding, but much less so to binding of SUMO2 [24].

In summary, this work provides the first clear demonstration of a requirement for non covalent SUMO-interaction motifs for SUMO E3 activity. Additionally, it appears that Pc2 may function differently from members of the PIAS family, which do not appear to require SIMs for E3 activity.

## Materials and Methods

### Cell Culture

COS1 cells were cultured in DMEM with 10% bovine growth serum. For live cell imaging and protein expression, COS1 cells were transfected with Fugene and with LipofectAMINE respectively, according to the manufacturers' instructions.

### Plasmids

Pc2, and CtBP constructs were expressed from modified pCMV5 plasmids with amino-terminal T7, Flag or His6 tags. Fluorescent protein fusions were expressed from modified pCS2 with amino-terminal fusions to eCFP or eYFP (Clontech), or to monomeric Cherry [47]. Pc2 amino-terminal fusions to a sumoylation consensus were created in pCS2, with an amino-terminal Flag tag and a carboxyl-terminal tag encoding VKPE, or VRPE. Pyruvate kinase sumoylation reporters were a kind gift from Ron Hay [45]. Point mutations and internal deletion mutants were generated by standard PCR techniques and verified by sequencing. SUMO expression constructs were created in modified pCMV5 and pCS2 vectors as above. FV and QFI mutations were introduced by PCR, in the context of SUMOs lacking the carboxyl-terminal tail after the di-glycine motif. The ΔGG mutants were generated by PCR, resulting in alteration of the two terminal glycines to alanines, with the carboxyl-terminal tail present.

### Cobalt Affinity Purification

Cells were lysed in 6M guanidine-HCl, 50 mM NaH_2_PO_4_ (pH 8.0), 10 mM Tris-HCl pH 8.0, 100 mM NaCl. His6 tagged proteins were bound to Talon Resin (Clontech) at room temperature. Resin was washed 3 times for 30 min with 8M Urea, 50 mM NaH_2_PO_4_ pH 7.0, 100 mM NaCl.

### Coimmunoprecipitation and Western Blotting

COS1 cells were lysed in PBS with 1% NP-40 (PBSN) and protease inhibitors (Roche). After centrifugation, lysates were immunoprecipitated with Flag-agarose (Sigma) for 2 h at 4°C. Beads were washed 3 times with PBS-N and proteins were separated by SDS-PAGE and transferred to Immobilon-P (Millipore). Proteins were visualized using ECL (Amersham).

### GST Binding Assays

GST-SUMO1 or GST-SUMO2 was purified from BL21 cells on glutathione agarose, as previously described [18]. Following elution with glutathione and dialysis, proteins (2 µg GST-SUMO per binding reaction) were rebound to glutathione agarose, blocked overnight with BSA, and incubated with lysates form transfected COS1 cells (in PBS with 0.1% Triton X-100 and protease inhibitors), or with His6-YFP-Pc2 fusions purified from bacteria. Following incubation with rocking at room temperature for 2 hours, the glutathione agarose was allowed to settle for 10 minutes and the supernatant removed. Beads were then washed 3 times with 1 ml of lysis buffer, by gentle mixing followed by settling at room temperature for 10 minutes. Bound Flag-tagged or His6-YFP-tagged Pc2 was detected by western blot, and GST-SUMO was visualized by Coomassie blue staining or western blot.

### Live Cell Imaging

For quantification, COS1 cells were plated into 4-well #1 cover-glass chamber slides (Nunc LabTek) and transfected using Fugene 6 (Roche). After 21-23 hr, cells were imaged with a Nikon Eclipse TE200 inverted fluorescence microscope with GFP, CFP, Rhodamine and DAPI filter sets, at 60x magnification using a water immersion lens (Nikon PlanApo 60x 1.20 WI). To quantify co-localization with Pc2, cells expressing Pc2 in sub-nuclear foci were selected, and scored for colocalization of the other fluorescent protein. In each experiment, at least 50 cells were scored for each combination, and the results of representative assays are shown. Black and white images were acquired using Openlab with a Hamamatsu digital camera (C4742-95) and colorized in Photoshop CS2 (9.0.2). The eYFP signal was false colored to green, and the eCFP or mCherry kept as white on black, as these colors retained the best contrast.
